# Efficient two-step multivariate random effects meta-analysis of individual participant data for longitudinal clinical trials using mixed effects models

**DOI:** 10.1186/s12874-019-0676-1

**Published:** 2019-02-14

**Authors:** Hisashi Noma, Kazushi Maruo, Masahiko Gosho, Stephen Z. Levine, Yair Goldberg, Stefan Leucht, Toshi A. Furukawa

**Affiliations:** 1 Department of Data Science, The Institute of Statistical Mathematics, 10-3 Midori-cho, Tachikawa, Tokyo, 190-8562 Japan; 2 Department of Biostatistics, Faculty of Medicine, University of Tsukuba, Ibaraki, Japan; 3 Department of Community Mental Health, University of Haifa, Haifa, Israel; 4 Department of Statistics, University of Haifa, Haifa, Israel; 5 Department of Psychiatry and Psychotherapy, Technische Universität München, Munich, Germany; 6 Department of Health Promotion and Human Behavior, Kyoto University Graduate School of Medicine / School of Public Health, Kyoto, Japan

**Keywords:** Individual participant data meta-analysis, Mixed effects models, Multivariate random effects model, Longitudinal studies, Heterogeneity

## Abstract

**Background:**

Mixed effects models have been widely applied in clinical trials that involve longitudinal repeated measurements, which possibly contain missing outcome data. In meta-analysis of individual participant data (IPD) based on these longitudinal studies, joint synthesis of the regression coefficient parameters can improve efficiency, especially for explorations of effect modifiers that are useful to predict the response or lack of response to particular treatments.

**Methods:**

In this article, we provide a valid and efficient two-step method for IPD meta-analyses using the mixed effects models that adequately addresses the between-studies heterogeneity using random effects models. The two-step method overcomes the practical difficulties of computations and modellings of the heterogeneity in the one-step method, and enables valid inference without loss of efficiency. We also show the two-step method can effectively circumvent the modellings of the between-studies heterogeneity of the variance-covariance parameters and provide valid and efficient estimators for the regression coefficient parameters, which are the primary objects of interests in the longitudinal studies. In addition, these methods can be easily implemented using standard statistical packages, and enable synthesis of IPD from different sources (e.g., from different platforms of clinical trial data sharing systems).

**Results:**

To assess the proposed method, we conducted simulation studies and also applied the method to an IPD meta-analysis of clinical trials for new generation antidepressants. Through the numerical studies, the validity and efficiency of the proposed method were demonstrated.

**Conclusions:**

The two-step approach is an effective method for IPD meta-analyses of longitudinal clinical trials using mixed effects models. It can also effectively circumvent the modellings of the between-studies heterogeneity of the variance-covariance parameters, and enable efficient inferences for the regression coefficient parameters.

## Background

In clinical trials for drug developments, the outcome variables often involve longitudinal repeated measurements. In these trials, the primary statistical analyses usually compare the responses from experimental treatments with a control treatment at the end of a follow-up period. However, during the follow-up period, dropouts or missing data often arise, and may seriously influence the validity and precision of the statistical inference. Conventionally, simple imputation methods such as LOCF (last observation carried forward) had been adopted to handle these problems, but these ad-hoc approaches have been known to provide biased results and possibly produce erroneous conclusions [1, 2]. Recently, regulatory guidelines concerning missing data problems in clinical trials have been issued [1, 3, 4], and these invalid practices are rapidly substituted by more sophisticated methods. The mixed effects model [5, 6], especially known as the MMRM (mixed effects model for repeated measures) [7], is one of these methods and has been widely adopted for primary analyses of recent clinical trials in drug developments, which allows for valid statistical inference based on the direct likelihood approach under the missing at random (MAR) assumptions [5, 6].

The mixed effects model are increasingly used in the context of evidence synthesis as well. Recent advances of clinical trials data sharing systems (e.g., ClinicalStudyDataRequest.com, the YODA project) have enabled evidence synthesis of large individual participant level datasets and explorations of effect modifiers that are useful to predict the response or lack of response to particular treatments, because the powers of treatment-by-covariate interaction tests increasingly gain using rich statistical information from multiple clinical trials [8, 9]. However, there are additional complexities in the individual participant data (IPD) meta-analysis to synthesize longitudinal trial datasets based on the mixed effects models, due to the longitudinal nature of the datasets. The most important issue is about addressing correlations among different time points in the repeated measurements and the between-studies heterogeneity. In the IPD meta-analyses of ordinary univariate regression models such as analysis of covariance (ANCOVA) models, the synthesizing analyses can be simply implemented by multivariate meta-analysis [10, 11], but for the mixed effects models, the between-studies heterogeneity should be adequately considered not only for the fixed effects coefficients, but also for the variance-covariance parameters, simultaneously. In addition, to use the statistical information among multiple trials maximally, we should consider the correlations among the estimators of model parameters to gain efficiency. In the IPD meta-analyses using mixed effects models, Ishak et al. [12] considered to aggregate only summarized data of each time point, but their methods do not use the correlation information. Also, Jones et al. [13] proposed to implement IPD meta-analyses of longitudinal data using mixed effects models, but their methods only assumed fixed effects models for the regression coefficient parameters, and the heterogeneity among the studies was not considered. In existing IPD meta-analyses, these problems have also not been addressed and fixed effects models that assume no heterogeneity have been usually used.

In this article, we provide a valid and efficient method for IPD meta-analyses using the mixed effects models that adequately address the between-studies heterogeneity using random effects models. We especially show the ordinary one-step methods have practical difficulties on computations and modellings of the heterogeneity, and we provide a two-step approach as an alternative effective procedure to overcome these problems. We will show that the two-step method can effectively circumvent the modellings of the between-studies heterogeneity of the variance-covariance parameters and provides valid and efficient estimators for the regression coefficient parameters, which are the primary objects of interests. In addition, these methods can be easily implemented using standard statistical software, and have advantages to synthesize IPD from different sources of clinical trial datasets (e.g., from different platforms of clinical trial data sharing systems). We also demonstrate the effectiveness of these methods via simulation studies and a real data application to an IPD meta-analysis of clinical trials for new generation antidepressants [14].

## Methods

### Mixed effects models for longitudinal data

We consider a meta-analysis of *N* longitudinal clinical trials with *n*_*i*_ individual participants within the *i*th trial (*i* = 1, … , *N*). Let *Y*_*ijt*_ be continuous repeated measurement outcome of *t*th time point of participant *j* in trial *i*, (*j* = 1, … , *n*_*i*_; *t* = 1, … , *T*), possibly to be missing at some points. Note that the time points planned to be measured the outcome variables are uncommon across the *N* trials, we can formally regard these unmeasured time point data as ‘missing’ without loss of generality.

At first, we discuss linear mixed effect models [5, 6] for the analysis of individual trials separately,

1 $$ {\boldsymbol{Y}}_{ij}={\boldsymbol{X}}_{ij}{\boldsymbol{\beta}}_i+{\boldsymbol{Z}}_{ij}{\boldsymbol{b}}_{ij}+{\boldsymbol{\varepsilon}}_{ij} $$

where ***Y***_*ij*_ ***=*** (*Y*_*ij*1_,  … , *Y*_*ijT*_)^*T*^ is a *T*-vector of repeated measurements for participant *j* in trial *i*, ***X***_*ij*_ ***=*** (***x***_*ij*1_,  … , ***x***_*ijT*_)^*T*^ is a *T* × *p* matrix of fixed effect explanatory covariates, ***β***_*i*_ is a *p*-vector of fixed regression coefficients, ***Z***_*ij*_ ***=*** (***z***_*ij*1_,  … , ***z***_*ijT*_)^*T*^ is a *T* × *q* matrix of random-effect covariates of participant *j* in trial *i* (usually a subset of ***X***_*ij*_), and ***b***_***ij***_ is a *q*-vector of random effects, assumed to follow a multivariate normal distribution ***b***_***ij***_***~****MVN*(**0**, ***D***_*i*_), where ***D***_***i***_ is the *q* × *q* covariance matrix of the random effects distribution. ***ε***_*ij*_ is a *T*-vector of random error, assumed to follow a multivariate normal distribution ***ε***_*ij*_***~****MVN*(**0**, **Σ**_*i*_), where **Σ**_*i*_ is the *T* × *T* covariance matrix of the random error distribution. The random effects ***b***_*ij*_ and the error ***ε***_*ij*_ are assumed to be independent, and thus the outcome variables among different participants are also independent. ***Y***_*ij*_ marginally follows *MVN*(***X***_*ij*_***β***_*i*_, ***V***_*ij*_), where ${\boldsymbol{V}}_{ij}={\boldsymbol{Z}}_{ij}\boldsymbol{D}{\boldsymbol{Z}}_{ij}^T+{\boldsymbol{\Sigma}}_i$. The model parameters can be estimated by maximum likelihood (ML) or restricted maximum likelihood (REML) methods [6]; we suppose to use the REML estimator here owing to its theoretical property [6, 15]. If missing outcome data are involved, these likelihood-based inference methods provide valid statistical inference results under MAR assumptions [5, 6], and thus the linear mixed models have been widely adopted in longitudinal data analyses. In addition, recently, owing to the developments of regulatory guidelines concerning missing data treatments in clinical trials [3, 4], the mixed effects models have been increasingly popular. Especially, an alternative parameterized model named as MMRM [7] has been adopted for primary analyses of many clinical trials, which directly models the marginal distribution of ***Y***_*ij*_’s and parameterize the covariance matrix ***V***_*ij*_ not specifying ***D*** and **Σ**_*i*_. The primary objects of interests are usually the fixed effects parameters, and we have rather little interests in the variance-covariance parameters in these clinical trials [16, 17]. Thus, through the re-parameterization to the marginal covariance matrix ***V***_*ij*_, possible model misspecifications concerning the random effects structures might be effectively circumvented in the statistical inference. The procedures of statistical analyses and their theoretical properties are identical between the two parameterized models, so we conduct the following discussions using the former model (1) in this article.

### IPD meta-analyses based on the mixed effects models

Based on the mixed effects model (1), IPD meta-analysis can be implemented in a straightforward manner. Jones et al. [13] proposed a fixed effects model with an assumption the regression coefficients ***β***_*i*_’s are common across the *N* trials using both of one-step and two-step approaches. However, the between-studies heterogeneity is an essential issue to be addressed in modern evidence synthesis researches [9], so the random effects models are preferred in general [9, 18]. While, there are several crucial problems to be addressed in applying them to the mixed effects models for IPD meta-analysis:

1. When we consider the heterogeneity across studies, we should adequately address the heterogeneities for both of the fixed effects and variance-covariance parameters in the synthesizing analyses. A straightforward approach would be to model these parameters as random effects, the joint random effects model for ***b***_*ij*_ and the components of ***V***_*ij*_. This strategy would also require complicated computations that cannot be implemented in standard statistical packages, and we should completely specify the statistical model without any misspecifications in order to assure the consistency of parameter estimation for fixed effects. Besides, we have little interests in the heterogeneities of variance-covariance parameters, i.e., the components of ***V***_*ij*_ in the longitudinal clinical trials [16, 17].

2. In most of meta-analyses of longitudinal trials, the time-points to be planned to collect outcome measurements are not common across studies. In the mixed effects model analyses, the time variables are often treated as factor variables (especially for the MMRM analyses [19]), and then all of the outcome variables at several time points are possibly missing (unmeasured) in some studies. In these cases, when we conduct a one-step random meta-analysis using standard statistical packages (e.g., PROC MIXED in SAS, lme4 [20] for R), the statistical model can be partially not identifiable, i.e., we cannot obtain the model estimates.

Given these problems, there are practical difficulties in implementing ordinary one-step IPD meta-analyses. Note that for the first problem, Jones et al. [13] proposed another approach to modelling the variance-covariance parameters as different parameters across studies in their fixed effects model framework, but these approaches are not implementable using standard packages. In addition, this problem has not been usually considered in many existing IPD meta-analyses, possibly due to its practical difficulties, and common variance-covariance structures have usually been assumed. Besides, the second issue can be resolved by making an original program that maximises the (restricted) likelihood function that assumes different regression functions across the studies (and possibly different random effects structures), but it requires usually large efforts in making the complicated original programs in case-by-case practices.

### Two-step marginal multivariate random effects analyses

To circumvent the complicated modelling problems and computational difficulties, we provide an alternative two-step approach for IPD meta-analyses based on the mixed effects models. Our proposition is to divide the model parameters into two components, fixed effects parameters and variance-covariance parameters, and to conduct synthesis analyses after obtaining study-specific aggregated statistics separately. Because we have little interests in the random effects in these clinical trials and the mixed effects models are typically designed to assess fixed effects [16, 17], here we specifically focus on the synthesis analyses for the fixed effects parameters. As noted later, the same method can be adapted to the synthesis analyses of the variance-covariance parameters. The computations are easily implementable, but we can show the resultant inference is valid and efficient, and that its theoretical properties do not depend on any heterogeneous structures of within-studies variance-covariance parameters.

We describe the procedure as follows.

- **Step-1.** Analyse the IPD from *N* longitudinal trials separately, using the mixed effects models (1), and obtain the REML estimates of the model parameters (${\widehat{\boldsymbol{\beta}}}_i,{\widehat{\boldsymbol{D}}}_i,{\widehat{\boldsymbol{\Sigma}}}_i$), *i* = 1, 2, … , *N*. In addition, obtain the variance-covariance matrix estimates of ${\widehat{\boldsymbol{\beta}}}_i$, ${\widehat{\boldsymbol{S}}}_i\left({\widehat{\boldsymbol{\beta}}}_i\right)$ for *i* = 1, 2, … , *N*.
- **Step-2.** Conduct synthesizing analyses for the regression coefficient parameters $\Big({\widehat{\boldsymbol{\beta}}}_i$, ${\widehat{\boldsymbol{S}}}_i\left({\widehat{\boldsymbol{\beta}}}_i\right)\Big)$ that are the primary objects of interests in these meta-analyses, dropping statistical information about the within-studies covariance parameters (${\widehat{\boldsymbol{D}}}_i,{\widehat{\boldsymbol{\Sigma}}}_i$),

2 $$ {\widehat{\boldsymbol{\beta}}}_i\sim MVN\left({\boldsymbol{\beta}}_i,{\widehat{\boldsymbol{S}}}_i\left({\widehat{\boldsymbol{\beta}}}_i\right)\right) $$

$$ {\boldsymbol{\beta}}_i\sim MVN\left(\boldsymbol{\mu}, \boldsymbol{\Omega} \right) $$

Inference concerning the model parameters (***μ***, **Ω**) can be conducted using the log likelihood or the restricted log likelihood functions [21, 22],

$$ \ell \left(\boldsymbol{\mu}, \boldsymbol{\Omega} \right)=-\frac{1}{2}\sum \limits_{i=1}^N\left\{\log \left|\boldsymbol{\Omega} +{\widehat{\boldsymbol{S}}}_i\left({\widehat{\boldsymbol{\beta}}}_i\right)\right|+{\left({\widehat{\boldsymbol{\beta}}}_i-\boldsymbol{\mu} \right)}^T{\boldsymbol{W}}_i\left({\widehat{\boldsymbol{\beta}}}_i-\boldsymbol{\mu} \right)+p\log 2\pi \right\}, $$

$$ {\ell}_{RL}\left(\boldsymbol{\mu}, \boldsymbol{\Omega} \right)=\ell \left(\boldsymbol{\mu}, \boldsymbol{\Omega} \right)-\frac{1}{2}\log \left|\sum \limits_{i=1}^N{\boldsymbol{W}}_i\right|+\frac{1}{2}p\log 2\pi $$

where $$ {\boldsymbol{W}}_i={\left(\boldsymbol{\Omega} +{\widehat{\boldsymbol{S}}}_i\left({\widehat{\boldsymbol{\beta}}}_i\right)\right)}^{-1} $$.

The computation of Step-1 is straightforwardly conducted using standard packages for linear mixed models, e.g., PROC MIXED in SAS, lme4 [20] for R. Also, Step-2 can be also simply implemented using existing packages for multivariate meta-analyses, e.g., mvmeta for Stata [23, 24] and R [25].

The resultant estimators of (***μ***, **Ω**) have the following properties.

#### Remark A

There are no efficiency losses regardless of dropping the within-studies variance-covariance information of (${\widehat{\boldsymbol{D}}}_i,{\widehat{\boldsymbol{\Sigma}}}_i$) in the multivariate meta-analysis models (2) for inferences of (***μ***, **Ω**), i.e., the ML and REML estimators of (***μ***, **Ω**) on the multivariate meta-analysis models (2) are the most efficient estimators, and their efficiency bound is equivalent to that of the one-step estimators for the completely specified correct model.

This property is assured because the ML and REML estimators of ***β***_*i*_ and (***D***_*i*_, **Σ**_*i*_) are orthogonal [6] in the sense of Cox and Reid [26], i.e., the non-diagonal submatrices of the information matrix of mixed model (1) are zero-matrices. Therefore, the statistical information of (${\widehat{\boldsymbol{D}}}_i,{\widehat{\boldsymbol{\Sigma}}}_i\Big)$ does not contribute to synthesizing ***β***_***i***_’s when we conduct one-step IPD meta-analyses under the completely specified correct model. Thus, fully efficient estimator of (***μ***, **Ω**) can be obtained via the two-step method above, even if the statistical information of (***D***_*i*_, **Σ**_*i*_) are dropped. This property is confirmed in the simulation studies in Section 3.

#### Remark B

When the heterogeneity for the variance-covariance parameters of (***D***_*i*_, **Σ**_*i*_) is modelled by a random effects model *G*(***D***, **Σ**), we can obtain the consistent and fully efficient estimator of (***μ***, **Ω**) using the two-step method under any distribution form of *G*(***D***, **Σ**).

Note this property is important that it assures the validity and optimality of the two-step method without modelling the random effects model *G*(***D***, **Σ**) explicitly, even if there exists substantial heterogeneity for the variance-covariance parameters. This property is assured because the ML and REML estimators of ***β***_*i*_ and (***D***_*i*_, **Σ**_*i*_) are orthogonal as the same arguments with Remark A. Besides, if we adopt the one-step approach, we should completely specify the joint random effects distributions of ***β***_*i*_ and (***D***_*i*_, **Σ**_*i*_) correctly to assure the consistency. These parametric assumptions are also not substantial objects of interests in the evaluations of treatment effects. Through the two-step approach, we can effectively circumvent the unnecessary additional complicated assumptions as well as the computational efforts in practices.

In addition, in IPD meta-analyses of longitudinal clinical trials, the time-points of outcome measurements are possibly uncommon across studies. As mentioned regarding the second issue in the previous section, when the time variables are often treated as factor variables (e.g., for the typical MMRM analyses [19]), the one-step approach has practical and computational difficulties.

#### Remark C

For synthesis of longitudinal clinical trials in which time-points of outcome measurements are possibly uncommon, we can apply the two-step approach formally via dropping the corresponding time-points variables in Step-1. Then, in Step-2, the corresponding components of ${\widehat{\boldsymbol{\beta}}}_i$ should be treated as missing outcomes variables.

The missing problems are common not only in two-step IPD meta-analysis, but in various multivariate meta-analysis, and have been extensively discussed in previous papers [21, 22]. Accordingly, in many software packages for multivariate meta-analysis [23–25], the standard modules can treat these partially missing outcomes adequately. Usually, these packages conduct available data analyses based on direct likelihood methods [23, 24].

In addition, in some cases, we can obtain the IPD for multiple clinical trials from different platforms of clinical trial databases. Recently, the clinical trial data sharing has been increasingly advanced [27, 28], and researchers can access the IPD of clinical trials for scientific research purposes, e.g., via ClinicalStudyDataRequest.com. However, in many platforms, the users cannot download the datasets and can only analyse on the remote accesses on the platform, in which case the IPD can only be analysed separately. In such cases, the one-step IPD meta-analyses cannot be conducted, but the two-step approaches can still be applied as valid and efficient procedure. In analysing longitudinal trial datasets, the two-step marginal synthesis method would be an effective strategy for evaluating treatment effects and interactions of the candidates of effect modifiers.

## Results

### Simulation studies

We conducted simulation studies to evaluate the validity and efficiency of the proposed method under practical IPD meta-analysis scenarios. In this context, validity corresponds to the accuracy of point estimation, standard error (SE) evaluation, and the coverage rate of the confidence interval (CI). In addition, the efficiency corresponds to the precision of estimation and the power of statistical test.

#### Simulation 1

First, we conducted simulations to evaluate the efficiency of two-step marginal multivariate meta-analysis approach. In particular, we would evaluate the equivalence of the two-step marginal method and the fully efficient one-step IPD meta-analysis. Simulation data were generated as three time-points longitudinal data, following the mixed effects model involving two-way interactions,

3 $$ {Y}_{ij t}={b}_{ij}+{\beta}_{i1}+{\beta}_{i2} trea{t}_{ij}+{\beta}_{i3} tim{e}_{2, ij}+{\beta}_{i4}{time}_{3, ij}+{\beta}_{i5}{z}_{ij}+{\beta}_{i6} trea{t}_{ij}\times tim{e}_{2, ij}+{\beta}_{i7} trea{t}_{ij}\times tim{e}_{3, ij}+{\beta}_{i8} tim{e}_{2, ij}\times {z}_{ij}+{\beta}_{i9} tim{e}_{3, ij}\times {z}_{ij}+{\beta}_{i10}{treat}_{ij}\times {z}_{ij}+{\varepsilon}_{ij t} $$

where *b*_*ij*_ is a random intercept for the *j*th participant of *i*th trial following *N*(0,4.5^2^) and *ε*_*ijt*_ is the random error for the *t*th visit of *j*th participant of *i*th trial following *N*(0,3.2^2^), *treat*_*ij*_ is a binary variable indicating treatment group. *time*_2, *ijt*_ and *time*_3, *ijt*_ are dummy variables indicating time-points of 2nd and 3rd visits, respectively (reference is the 1st visit). The parameter settings were mimicked to the Furukawa et al. [14]‘s example in Section 4. *z*_*ij*_ is a covariate variable that is a candidate to be an effect modifier, which is a measurement that is associated with response or lack of response to a particular therapy. The two-way interaction terms represent the potential usefulness of *z*_*ij*_ as an effect modifier. Following the mixed effects model (3), we generated *N* longitudinal clinical trial datasets, for *N* = 5, 10, 15, and 20. In addition, the number of participants of each trial was generated from a discrete uniform distribution on [50, 500].

In these first simulation studies, the primary purpose is to assess the validity and efficiency of the two-step approach provided in Section 2. Also, we would evaluate the equivalence of the two-step marginal method and the fully efficient one-step IPD meta-analysis. At first, to circumvent the discussions to be complicated, we assumed there was no heterogeneity for ***β***_*i*_ and (***D***_*i*_, **Σ**_*i*_) among the *N* trials, which is a special case of the fixed effects models considered by Jones et al. [13]. In other words, all ***β***_*i*_ (*i* = 1, … , *N*) are equal to the grand mean ***μ***. The parameter settings of ***μ*** were also mimicked to the Furukawa et al. [14], *μ*_1_ =  − 0.04, *μ*_2_ =  − 0.14, *μ*_3_ = 1.56, *μ*_4_ = 0.83, *μ*_5_ =  − 0.46, *μ*_6_ = 0.57, *μ*_7_ = 0.14, *μ*_8_ = 0.15,  *μ*_9_ = 0.05, *μ*_10_ =  − 0.06. Under these settings, we conducted 10,000 simulations, and we analyzed the simulated datasets using the one-step method by the correctly specified mixed effects model and the two-step multivariate meta-analysis approach in Section 2.

The results are presented in Table 1. We presented empirical means and SE of the regression coefficient estimates, means of the SE estimates ($\widehat{\mathrm{SE}}$), empirical coverage rates of the 95% CIs, and empirical powers for the tests of individual regression coefficients. The contexts of the results are too rich, so we present the ones for selected coefficients, *μ*_2_, *μ*_4_, *μ*_5_, *μ*_7_, *μ*_9_, *μ*_10_. The results for the remaining parameters were similar. These results consistently showed that the fully efficient one-step approach was equivalent to the marginal two-step approach under all of the scenarios. Both the mean and SE of 10,000 estimates were mostly the same, and the SE estimate could accurately estimate the actual SE of the estimates. In addition, coverage rates of 95% CIs were accurate and the power of the two approaches were equivalent. As a whole, the two-step approach was shown to provide accurate results without loss of efficiency, regardless of dropping the variance-covariance information. The efficiency was also retained for the interaction tests.

**Table 1:** Results for the simulation 1: fully efficient one-step approach and the two-step marginal multivariate meta-analysis approach were compared

	*N* = 5		*N* = 10		*N* = 15		*N* = 20	
	One-step	Two-step	One-step	Two-step	One-step	Two-step	One-step	Two-step
*μ*_2_ = 0.14								
Mean	0.135	0.135	0.146	0.146	0.135	0.136	0.130	0.130
SE	0.866	0.867	0.612	0.613	0.495	0.496	0.434	0.435
$\widehat{\mathrm{SE}}$	0.867	0.866	0.608	0.606	0.495	0.494	0.428	0.427
Coverage Rate	0.949	0.950	0.948	0.947	0.949	0.947	0.950	0.948
Power	0.052	0.053	0.056	0.058	0.058	0.059	0.071	0.070
*μ*_4_ = 0.83								
Mean	0.827	0.826	0.836	0.836	0.828	0.829	0.825	0.824
SE	0.415	0.416	0.287	0.287	0.236	0.236	0.206	0.205
$\widehat{\mathrm{SE}}$	0.410	0.410	0.287	0.287	0.234	0.234	0.202	0.202
Coverage Rate	0.950	0.950	0.951	0.951	0.948	0.947	0.939	0.941
Power	0.533	0.533	0.825	0.825	0.936	0.937	0.978	0.978
*μ*_5_ = −0.46								
Mean	−0.461	−0.461	−0.460	−0.460	−0.460	−0.460	−0.461	−0.461
SE	0.032	0.032	0.023	0.023	0.018	0.018	0.016	0.016
$\widehat{\mathrm{SE}}$	0.032	0.032	0.022	0.022	0.018	0.018	0.016	0.016
Coverage Rate	0.949	0.948	0.949	0.947	0.953	0.955	0.951	0.949
Power	1.000	1.000	1.000	1.000	1.000	1.000	1.000	1.000
*μ*_7_ = 0.14								
Mean	0.139	0.139	0.141	0.141	0.143	0.143	0.144	0.144
SE	0.178	0.178	0.122	0.122	0.100	0.100	0.088	0.088
$\widehat{\mathrm{SE}}$	0.176	0.176	0.123	0.123	0.100	0.100	0.087	0.087
Coverage Rate	0.948	0.947	0.949	0.949	0.949	0.949	0.940	0.942
Power	0.127	0.128	0.211	0.215	0.295	0.297	0.382	0.381
*μ*_9_ = 0.05								
Mean	0.050	0.050	0.050	0.050	0.050	0.050	0.050	0.050
SE	0.020	0.020	0.014	0.014	0.011	0.011	0.010	0.010
$\widehat{\mathrm{SE}}$	0.020	0.020	0.014	0.014	0.011	0.011	0.010	0.010
Coverage Rate	0.947	0.947	0.951	0.951	0.949	0.952	0.941	0.940
Power	0.734	0.735	0.947	0.947	0.995	0.995	0.999	0.999
*μ*_10_ = −0.06								
Mean	−0.060	−0.060	−0.060	−0.060	−0.060	−0.060	−0.059	−0.059
SE	0.042	0.042	0.030	0.030	0.024	0.024	0.021	0.021
$\widehat{\mathrm{SE}}$	0.042	0.042	0.029	0.029	0.024	0.024	0.021	0.021
Coverage Rate	0.949	0.949	0.948	0.947	0.949	0.950	0.946	0.945
Power	0.301	0.302	0.540	0.543	0.700	0.701	0.811	0.811

#### Simulation 2

Second, we conducted other simulations to evaluate the validity of the two-step random effects multivariate meta-analysis approaches under substantial heterogeneity of ***β***_*i*_ as well as (***D***_*i*_, **Σ**_*i*_)**,** among *N* longitudinal trials. We considered the mixed effects models (3) for individual trials, and additionally considered heterogeneity of ***β***_*i*_ and (***D***_*i*_, **Σ**_*i*_). We considered a compound symmetry structure for **Ω**, which assumes a common variance among all of the components of ***β***_*i*_, *β*_*ik*_~*N*(*μ*_*k*_, *τ*^2^), *k* = 1, … , 10, where *τ*^2^ = 0.10, 0.20. We assumed no correlations among *β*_*ik*_s, and also assumed heterogeneity among the random effects distributions of *b*_*ij*_~*N*(0, *ζ*^2^), where *ζ* is generated from a continuous uniform distribution on [2.5, 9.0]. We considered two settings: (a) outcome variables are measured on all three time-points, and (b) outcome variables are measured on two time-points for 40% of trials and on three time-points for the remaining 60% trials.

Under these conditions, the two-step approach can be easily implemented using standard statistical packages. Again, note that the two-step method does not require modelling of the heterogeneity structures of (***D***_*i*_, **Σ**_*i*_)**,** and the validity and efficiency of the inference are assured under any heterogeneous structures of (***D***_*i*_, **Σ**_*i*_). Besides, to implement valid inference using one-step approach, we should adopt the correct statistical model involving the random effects distributions for both of ***β***_*i*_ and (***D***_*i*_, **Σ**_*i*_). One ad-hoc approach uses a Bayesian hierarchical modelling involving random effects models, but it requires complicated computations using special software (e.g., OpenBUGS [29]).

The simulation results are presented in Tables 2 and 3, which correspond to the settings (a) and (b) respectively. We conducted 10,000 simulations and provided the same outputs as Table 1. We considered 6 scenarios, setting *N* = 5, 10, or 15 and *τ*^2^ = 0.10 or 0.20. The results also showed unbiasedness of the two-step estimates under all of the scenarios. In addition, the validities of SE estimation and CIs (inversely, the sizes of the corresponding statistical tests) could be confirmed. Again, note that we only took parametric assumptions for the random effects distribution of ***β***_*i*_ and no restrictive assumptions concerning the heterogeneity of (***D***_*i*_, **Σ**_*i*_). The validity of the two-step method was demonstrated under the flexible and weak assumptions. The efficiency is not compared with those of the one-step methods here, but the equivalence is theoretically assured (Remark B).

**Table 2:** Results for the simulation 2(a): outcome variables were measured on all three time-points

	*N* = 5		*N* = 10		*N* = 15	
	*τ*^2^ = 0.10	*τ*^2^ = 0.20	*τ*^2^ = 0.10	*τ*^2^ = 0.20	*τ*^2^ = 0.10	*τ*^2^ = 0.20
*μ*_2_ = 0.14						
Mean	0.141	0.144	0.141	0.144	0.145	0.143
SE	0.694	0.709	0.694	0.709	0.564	0.565
$\widehat{\mathrm{SE}}$	0.680	0.692	0.680	0.692	0.553	0.562
Coverage Rate	0.946	0.947	0.946	0.947	0.947	0.950
Power	0.061	0.057	0.061	0.057	0.059	0.057
*μ*_4_ = 0.83						
Mean	0.828	0.831	0.828	0.831	0.832	0.833
SE	0.306	0.323	0.306	0.323	0.252	0.268
$\widehat{\mathrm{SE}}$	0.307	0.325	0.307	0.325	0.251	0.265
Coverage Rate	0.951	0.950	0.951	0.950	0.948	0.947
Power	0.773	0.725	0.773	0.725	0.912	0.877
*μ*_5_ = −0.46						
Mean	−0.459	−0.458	−0.459	−0.458	−0.461	−0.460
SE	0.104	0.143	0.104	0.143	0.084	0.117
$\widehat{\mathrm{SE}}$	0.103	0.143	0.103	0.143	0.084	0.117
Coverage Rate	0.944	0.947	0.944	0.947	0.949	0.949
Power	0.992	0.890	0.992	0.890	1.000	0.976
*μ*_7_ = 0.14						
Mean	0.142	0.141	0.142	0.141	0.138	0.140
SE	0.163	0.193	0.163	0.193	0.134	0.157
$\widehat{\mathrm{SE}}$	0.163	0.192	0.163	0.192	0.133	0.157
Coverage Rate	0.947	0.948	0.947	0.948	0.947	0.951
Power	0.140	0.115	0.140	0.115	0.181	0.146
*μ*_9_ = 0.05						
Mean	0.051	0.050	0.051	0.050	0.048	0.050
SE	0.101	0.140	0.101	0.140	0.082	0.116
$\widehat{\mathrm{SE}}$	0.100	0.141	0.100	0.141	0.082	0.116
Coverage Rate	0.944	0.945	0.944	0.945	0.947	0.947
Power	0.091	0.071	0.091	0.071	0.091	0.076
*μ*_10_ = −0.06						
Mean	−0.061	−0.058	−0.061	− 0.058	− 0.060	−0.061
SE	0.106	0.145	0.106	0.145	0.087	0.119
$\widehat{\mathrm{SE}}$	0.105	0.145	0.105	0.145	0.086	0.119
Coverage Rate	0.945	0.944	0.945	0.944	0.945	0.947
Power	0.096	0.074	0.096	0.074	0.110	0.084

**Table 3:** Results for the simulation 2(b): outcome variables were measured on two time-points for 40% trials and on three time-points for 60% trials

	*N* = 5		*N* = 10		*N* = 15	
	*τ*^2^ = 0.10	*τ*^2^ = 0.20	*τ*^2^ = 0.10	*τ*^2^ = 0.20	*τ*^2^ = 0.10	*τ*^2^ = 0.20
*μ*_2_ = 0.14						
Mean	0.140	0.142	0.144	0.136	0.136	0.139
SE	1.026	1.026	0.693	0.702	0.483	0.559
$\widehat{\mathrm{SE}}$	0.989	1.004	0.681	0.690	0.475	0.561
Coverage Rate	0.947	0.950	0.948	0.946	0.948	0.953
Power	0.057	0.052	0.058	0.058	0.061	0.053
*μ*_4_ = 0.83						
Mean	0.825	0.822	0.823	0.828	0.873	0.836
SE	0.546	0.584	0.377	0.404	0.283	0.329
$\widehat{\mathrm{SE}}$	0.537	0.574	0.376	0.402	0.257	0.328
Coverage Rate	0.948	0.950	0.950	0.947	0.907	0.947
Power	0.347	0.316	0.591	0.548	0.892	0.717
*μ*_5_ = −0.46						
Mean	−0.459	−0.459	−0.460	−0.459	−0.460	−0.461
SE	0.147	0.204	0.103	0.143	0.073	0.118
$\widehat{\mathrm{SE}}$	0.143	0.200	0.102	0.143	0.073	0.117
Coverage Rate	0.931	0.932	0.944	0.944	0.944	0.945
Power	0.874	0.625	0.994	0.890	1.000	0.974
*μ*_7_ = 0.14						
Mean	0.145	0.145	0.136	0.135	0.138	0.141
SE	0.295	0.359	0.207	0.245	0.147	0.202
$\widehat{\mathrm{SE}}$	0.291	0.346	0.206	0.245	0.145	0.201
Coverage Rate	0.943	0.936	0.947	0.950	0.946	0.949
Power	0.088	0.083	0.105	0.082	0.167	0.110
*μ*_9_ = 0.05						
Mean	0.051	0.052	0.051	0.055	0.049	0.050
SE	0.185	0.263	0.133	0.184	0.093	0.151
$\widehat{\mathrm{SE}}$	0.178	0.252	0.128	0.181	0.091	0.149
Coverage Rate	0.925	0.927	0.935	0.940	0.941	0.943
Power	0.086	0.080	0.084	0.070	0.089	0.070
*μ*_10_ = −0.06						
Mean	−0.062	− 0.063	− 0.060	− 0.060	− 0.060	− 0.059
SE	0.150	0.205	0.106	0.146	0.075	0.118
$\widehat{\mathrm{SE}}$	0.147	0.203	0.105	0.145	0.074	0.118
Coverage Rate	0.934	0.937	0.942	0.943	0.947	0.948
Power	0.084	0.074	0.096	0.079	0.130	0.083

### Applications to IPD meta-analysis for new generation antidepressants

To illustrate our method, we analyzed the dataset from an IPD meta-analysis for new generation antidepressants by Furukawa et al. [14] The authors conducted IPD meta-analysis from four placebo-controlled, double-blinded randomized clinical trials (1482 participants) for patients undergoing acute phase treatment for major depression. The outcome variable was the change score on the 17-item Hamilton Rating Scale for Depression (HRSD) or the Montgomery-Asberg Depression Rating Scale (MADRS). In their analyses, the latter was converted into the former using the conversion algorithm based on the item response theory [30]. In particular, the authors investigated whether the baseline depression severity modifies the efficacy of the antidepressants using IPD meta-analysis. The outcome measurements were repeatedly measured at several time-points in the four trials, but the time-points at which the outcome variables were planned to be measured were not common across the four trials.

We considered the following mixed effects model on 1st, 2nd, 4th and 6th weeks involving two-way interaction terms based on the model (1),

$$ {Y}_{ij t}={b}_{ij}+{\beta}_{i1}+{\beta}_{i2} trea{t}_{ij}+{\beta}_{i3}{week}_{1, ij}+{\beta}_{i4}{week}_{2, ij}+{\beta}_{i5}{week}_{4, ij}+{\beta}_{i6}{z}_{ij}+{\beta}_{i7} trea{t}_{ij}\times {week}_{1, ij}+{\beta}_{i8} trea{t}_{ij}\times {week}_{2, ij}+{\beta}_{i9} trea{t}_{ij}\times {week}_{4, ij}+{\beta}_{i10}{week}_{1, ij}\times {z}_{ij}+{\beta}_{i11}{week}_{2, ij}\times {z}_{ij}+{\beta}_{i12}{week}_{4, ij}\times {z}_{ij}+{\beta}_{i13}{treat}_{ij}\times {z}_{ij}+{\varepsilon}_{ij t} $$

where *week*_1, *ij*_, *week*_2, *ij*_ and *week*_4, *ij*_ are dummy variables for the time points (reference: 6th week). We first attempted a one-step random effects meta-analysis that modelled all of the regression coefficients as random effects among the 4 trials, but the REML estimate was not computable using standard statistical packages. There might also be between-studies heterogeneity for the variance-covariance parameters, which was not modelled in this analysis. Thus, we provided the results of a one-step fixed effects meta-analysis only consider correlations of repeated measured outcomes among different time points within individual participants. The REML estimates, 95% CI, and *P*-values are presented in Table 4.

**Table 4:** Results of the IPD meta-analysis of 4 clinical trials for new generation antidepressants

	One-step method (Fixed effects model)				Two-step method (Fixed effects model)				Two-step method (Random effects model)			
	Estimate	95%CI		*P*-value	Estimate	95%CI		*P*-value	Estimate	95%CI		*P*-value
*μ*_1_	2.100	−0.407	4.606	0.101	1.353	−0.884	3.590	0.236	−0.040	−2.313	2.234	0.973
*μ*_2_	−0.029	−2.813	2.755	0.984	0.284	−2.183	2.750	0.822	0.193	−2.311	2.697	0.880
*μ*_3_	2.829	1.574	4.084	< 0.001	2.790	1.537	4.043	< 0.001	2.933	1.660	4.207	< 0.001
*μ*_4_	1.865	0.604	3.125	0.004	1.812	0.553	3.072	0.005	1.576	0.297	2.856	0.016
*μ*_5_	0.979	−0.289	2.247	0.130	0.985	−0.282	2.252	0.128	0.846	−0.442	2.134	0.198
*μ*_6_	−0.547	−0.663	−0.430	< 0.001	−0.511	−0.615	−0.407	< 0.001	−0.456	−0.571	−0.341	< 0.001
*μ*_7_	1.022	0.487	1.558	< 0.001	1.019	0.486	1.551	< 0.001	1.081	0.544	1.618	< 0.001
*μ*_8_	0.551	0.014	1.088	0.044	0.536	0.002	1.071	0.049	0.577	0.038	1.115	0.036
*μ*_9_	0.110	−0.431	0.651	0.689	0.104	− 0.435	0.642	0.705	0.141	−0.402	0.684	0.610
*μ*_10_	0.193	0.137	0.249	< 0.001	0.196	0.140	0.251	< 0.001	0.189	0.116	0.261	< 0.001
*μ*_11_	0.138	0.082	0.194	< 0.001	0.141	0.085	0.197	< 0.001	0.150	0.077	0.222	< 0.001
*μ*_12_	0.045	−0.011	0.102	0.117	0.045	−0.011	0.102	0.115	0.049	−0.024	0.123	0.185
*μ*_13_	−0.046	−0.175	0.082	0.479	−0.063	−0.177	0.051	0.278	−0.066	−0.190	0.058	0.294

Next, we conducted IPD meta-analyses using the two-step approach based on the REML estimates of the fixed effects coefficients of the 4 trials. In the multivariate random effects meta-analysis models, we considered several assumptions for between-studies variance-covariance structures **Ω**, and the fitting of the candidate models was evaluated using the Bayesian information criterion (BIC) [31]. The best fitting model was the compound symmetry model (BIC = 125.73), whereas for the fixed effects model, BIC = 316.89.

In Table 3, we also provided the results of two-step multivariate meta-analysis of the fixed effects model and the random effects model using the compound symmetry model. In the random effects model, the between-studies SD estimate was 0.047 and the between-studies correlation coefficient was 0.064. The results for the fixed effects model analyses using one-step and two-step approaches were consistent, which was expected as described in Remark A. However, note that even if there was between-studies heterogeneity for the variance-covariance parameters, the two-step approach provides valid inference results. Besides, comparing the fixed and random effects two-step analyses, the coefficient estimates were somewhat different. In addition, the SE estimates of the fixed effects coefficients were larger for the random effects model, indicating that uncertainty might be larger when using this model. Considering the simulation results, even when there is substantial heterogeneity in ***β***_*i*_s, the two-step random effects approach provides valid inference results. The interaction tests of *β*_*i*13_ were not significant by significance level of 5% for any of the three models, consistent with the original analyses of Furukawa et al. [14], but these tests should be the most powerful under the assumption that the statistical models were correct.

## Discussion

IPD meta-analysis is becoming increasingly popular in recent medical studies, especially as an effective approach for exploring effect modifiers for precision medicine [8]. Given recent trends in clinical trial data sharing [27, 28], the practical values of these methodologies are bound to increase in future studies. The efficiencies of the one-step and two-step approaches have also been discussed for IPD meta-analyses based on the conventional ANCOVA, logistic and Cox regression models [32]. Besides, as noted above, IPD meta-analyses using the mixed effects models involve more complicated problems, which should address the heterogeneity of variance-covariance structures of random effects distributions. In this paper, we presented a flexible two-step method that can effectively circumvent these difficulties, and demonstrated its validity and efficiency.

In the simulation studies, the coverage rates of 95% CIs for the random effects model retained nearly at the nominal level, but previous studies showed that SE are possibly underestimated under small *N* settings in random effects meta-analyses, and the standard CIs can have serious undercoverage properties [33–36]. In the simulation studies, the proper coverage properties are possibly caused by the adoption of a compound symmetry structure for the heterogeneity covariance matrix **Ω**. In multivariate meta-analyses, when the heterogeneity variances are assumed to be common across different outcomes (this assumption is often adopted in network meta-analysis [37, 38]), the statistical information across the different outcomes can be shared for the common parameter estimation, so the accuracy is generally gained. When we adopted different variance assumptions for **Ω**, the CIs had undercoverage property under small *N* setting (data not shown). Note that several improved methods for addressing the undercoverage property have been developed, e.g., the Kenward-Roger method [39] and the Bartlett-type corrections [40].

In this paper, we discussed the linear mixed effects models for continuous Gaussian outcome variables, but the two-step approach can be similarly adapted to the generalized linear mixed effects models for non-Gaussian outcome variables [41] and the frailty models for survival outcome variables [42]. For these outcome variables, it should be noted that there are possibly correlations between the estimators of fixed effects coefficients and variance-covariance parameters. Thus, the two-step analyses may cause losses of efficiency on the inferences. However, the two-step approach does not require parametric assumptions for heterogeneities for the within-studies variance-covariance parameters, and validity of the inference is generally assured. In addition, this method can be easily implemented using standard statistical packages. Therefore, the two-step marginal approach would also be an effective solution for these settings. Further research is warranted to evaluate the expansion of the two-step model to these settings.

## Conclusions

The two-step approach is an effective method for synthesizing the regression coefficient parameters efficiently for IPD meta-analyses of longitudinal clinical trials. Using this method, modelling of heterogeneities of the within-studies variance-covariance parameters can be effectively circumvented without additional parametric assumptions, which are not of primary interests in the analyses of these clinical trials [16, 17, 19]. If we adopt the one-step approach to modelling the heterogeneity, complicated modelling is required and the validity is possibly violated if the adopted model is misspecified. Based on the relaxed assumptions of the two-step approach, we can implement efficient inferences for the treatment effects and interactions. In addition, the two-step approach has advantages for implementations because it can be easily performed using standard statistical packages [23–25]. It can also be effectively applied to IPD meta-analyses using clinical trials data sharing systems in which individual trials can only be analyzed separately.

## Abbreviations

ANCOVA: analysis of covariance
BIC: Bayesian information criterion
CI: confidence interval
HRSD: Hamilton Rating Scale for Depression
IPD: individual participant data
MADRS: Montgomery-Asberg Depression Rating Scale
MAR: missing at random
ML: maximum likelihood
MMRM: mixed effects model for repeated measures
REML: restricted maximum likelihood
SE: standard error
